# Three Dimensional Distribution of Sensitive Field and Stress Field Inversion of Force Sensitive Materials under Constant Current Excitation

**DOI:** 10.3390/s18030722

**Published:** 2018-02-28

**Authors:** Shuanfeng Zhao, Min Liu, Wei Guo, Chuanwei Zhang

**Affiliations:** School of Mechanical Engineering, Xi’an University of Science and Technology, Xi’an 710054, China; liumin5432@163.com (M.L.); Guow@xust.edu.cn (W.G.); zhangcw@xust.edu.cn (C.Z.)

**Keywords:** three-dimensional force sensor, conductive silicone rubber, tactile sensor

## Abstract

Force sensitive conductive composite materials are functional materials which can be used as the sensitive material of force sensors. However, the existing sensors only use one-dimensional electrical properties of force sensitive conductive materials. Even in tactile sensors, the measurement of contact pressure is achieved by large-scale arrays and the units of a large-scale array are also based on the one-dimensional electrical properties of force sensitive materials. The main contribution of this work is to study the three-dimensional electrical properties and the inversion method of three-dimensional stress field of a force sensitive material (conductive rubber), which pushes the application of force sensitive material from one dimensional to three-dimensional. First, the mathematical model of the conductive rubber current field distribution under a constant force is established by the effective medium theory, and the current field distribution model of conductive rubber with different geometry, conductive rubber content and conductive rubber relaxation parameters is deduced. Secondly, the inversion method of the three-dimensional stress field of conductive rubber is established, which provides a theoretical basis for the design of a new tactile sensor, three-dimensional stress field and space force based on force sensitive materials.

## 1. Introduction

Over the past decades, conductive polymer materials have attracted a great deal of attention due to their potential applications, especially in the field of intelligent artificial skin, tactile sensing and intelligent sensing gloves [1,2,3,4,5]; Conductive silicone is a conductive polymer composite material that is the core material of flexible pressure sensors. There are lots of reasons to demonstrate that conductive silicone is suitable for flexible pressure sensors, which include: (1) the elasticity and softness of silicone rubber are similar to that of human skin and muscle and it has good biocompatibility, chemical stability and no toxicity, so it can be used to make wearable sensors [6,7,8]; (2) it has larger deformation, so it can be used in the area of flexible sensing and so on [9,10]; (3) high sensitivity and resolution in detection [11]; (4) low cost [12]; (5) the chemical properties of silicon compounds are very stable so its sensor products have longer life than those made from other materials [13,14]; (6) it has good processing performance, shaping easily, and other advantages, so it can be used to produce sensor devices of various shapes through hot air vulcanization molding extrusion, mold processing, calendar molding, and other methods [15,16,17,18].

Conductive silicone rubber is usually prepared by adding conductive fillers to the silicone rubber [19]. The commonly used conductive fillers are metal series conductive fillers or carbon series conductive fillers. Metal series materials are important fillers of conductive silicone rubber because of their excellent conductivity, the stable chemical properties of the filled polymer and the persistent conductive properties of the material [20]. In recent years, the silver plating technology has overcome the shortcomings of high density and easy oxidation of metal series conductive fillers. However, it is also accompanied by defects of the filler coating that easily falls off [21]. The carbon series conductive filler resources are abundant and they are not easily oxidized when being used, so the physical and mechanical properties of the polymer materials are ensured. Carbon black, which is cheap, can be made from a wide range of raw materials, and has small unit density, is easy to process and shape, gives good dispersions, good effects on the rubber compound, and chemical stability, is generally selected as the carbon-based conductive filler [22].

The second area of research is the mechanical and electrical mechanism of conductive silicone rubber. The study of the conductive properties of conductive silica gel shows that the essence of the conductive process in conductive silicone is the directional movement of carriers in an electric field. The conductive channels formed by macromolecule inter-chains and sufficient numbers of carriers are two important factors in conducting polymer conductivity [23]. The conductivity of conductive silica can be explained by the macro mechanism and the micro mechanism [24]. When the content of conductive particles is low, the conductive particles occupy less volume in the basic body of the silica gel polymer, the resistivity of basic body is large and is basically acts as insulation, so the resistivity of conductive polymer filled with conductive powder is greater [25]. As the number of conductive particles increases, the percentage in the main material increases, the distance between particles decreases, the motion of electrons in the particles increases and the transition probability increases, so the conductive mechanism can be explained by the microscopic quantum effect [26]. As the amount of conductive filler increases, the number of particle collisions increases, and can easily form a continuous conductive chain, which explains the macroscopic conductive channel theory. When a conductive network is formed in the compound, the conductivity is basically stable, and further addition of conductive particles has little effect on its conductivity [27].

The third kind of research is the application of conductive silica in various functional sensing applications. In one-dimensional array tactile sensor research, pressure-sensitive conductive silicone has been widely used in many fields such as artificial muscle [28,29,30], intelligent clothing [31], active wings [32] and so on. For example, the force sensitive characteristics of conductive silica gel are applied by some researchers. They weave together conductive rubber sheet and ordinary gloves to get a flexible tactile glove that can sense the action of the human hand [33]. Conductive rubber has more research results in one-dimensional force flexible tactile sensors because of its excellent force sensitive performance. The conductive rubber is limited by its preparation process (such as rubber basic body, filler, additives, vulcanization, etc.). Its application in multidimensional tactile sensors is relatively limited, but it has begun to develop from one-dimensional force sensing to multidimensional force perception. In recent years, researchers have been trying to make use of force sensitive conductive rubber to make the flexible touch intelligent skins for intelligent robots [34] and have obtained certain research breakthroughs. Arrays of parallel and stretchable electrodes and piezoresistive elastomeric composites have been commonly utilized to obtain the tactile information from large areas [35]. However, multi-directional tactile information requires additional stretchable electrodes in a limited space, which increases the difficulties in the fabrication process.

In this paper, a method for inversion of the stress distribution in large deformation conductive silicone rubber is proposed, which provides a theoretical basis for the design of cheap three-dimensional force flexible large deformation sensors.

The key contribution of this work is to establish a three-dimensional electrical characteristic model of conductive silicon rubber under large deformation stress state and then to inverse the distribution of its stress field, which will apply the electrical characteristics of conductive silicone rubber from one dimension to three-dimensional. Firstly, the mathematical model of conductive rubber current field distribution under constant force is established by using effective medium theory and the current field distribution model of conductive rubber under various geometric shapes, abundance of metals, conductive rubber relaxation parameters is deduced. Secondly, the inversion method of the three-dimensional stress field of conductive rubber is established, and the influence of electrode shape, electrode number and electrode position on the three-dimensional stress inversion mechanism under different geometric shapes is studied, which provides a theoretical basis for the design of a new tactile sensor, three-dimensional stress field and space force based on force sensitive materials.

## 2. Theory

### 2.1. Conductivity Mechanism

There are three mechanisms in the conductive mechanism of conductive silicone, which include conductive channel [36], tunnel effect [37] and field emission mechanism [38], but the contribution of each one differs under different conditions. When the content of conductive filler is high, the spacing of conductive particles is short, so it is easy to form conductive channel chains. At this time, the conduction mode is mainly via the conductive channel, and the seepage mechanism is obvious. When the content of conductive filler is less and the voltage is low, the distance between conductive particles is larger, it is not easy to form chains of conductive channels, and thermal vibration induces electronic transitions to form tunneling currents, so the tunnel effect theory plays a leading role. When the content of conductive filler is less, and the voltage is higher, the electric field of conductive particles is very strong and the field emission effect theory plays a major role.

**Figure 1:**
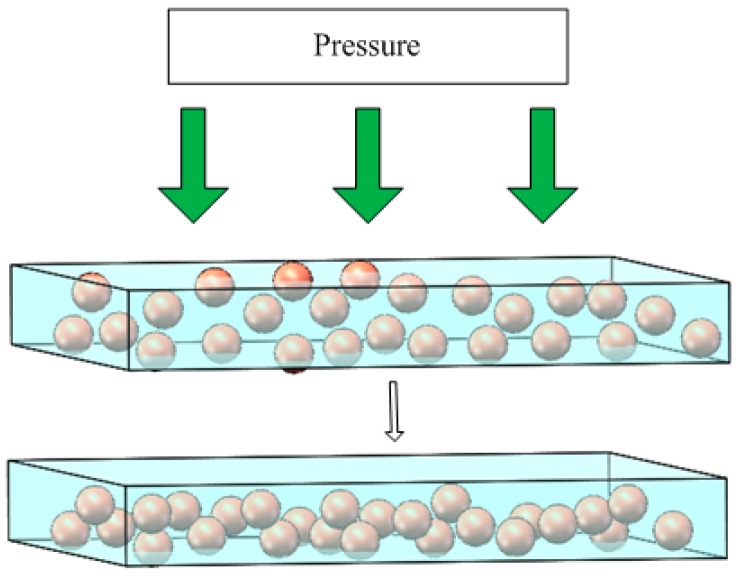
The formation of conductive channels.

The conductive mechanism of conductive silica gel should be the result of the interaction of conduction channel theory and tunneling current theory. In short, the actual conductive mechanism of conductive silica is rather complex, and the final result will be the comprehensive effect of various conductive mechanisms. Conductive particles with different sizes and shapes are evenly distributed in the insulation matrix. When the volume fraction of the filler reaches a critical value, the conductive particles or aggregates contact each other, forming a large number of conductive network channels, and the resistivity drops sharply. Figure 1 shows the contact of conductive particles with each other to form conductive channels.

McLachlan et al. proposed a general effective medium model [39] to account for the conductive mechanism of composite polymeric materials with particle fillers. The general effective medium equation is:

(1) $$\frac{\left(1-\phi \right)\left(\sigma_{1}^{1/\tau }-\sigma_{m}^{1/\tau }\right)}{\sigma_{1}^{1/\tau }+\left[\left(1-\phi_{c}\right)\phi_{c}\right]\sigma_{m}^{1/\tau }}+\frac{\phi \left(\sigma_{h}^{1/\tau }-\sigma_{m}^{1/\tau }\right)}{\sigma_{h}^{1/\tau }+\left[\left(1-\phi_{c}\right)\phi_{c}\right]\sigma_{m}^{1/\tau }}=0$$

In the formula: ϕ is the volume fraction of conductive filler; $\phi_{c}$ is the critical percolation threshold; $\sigma_{1}$ is the conductivity of the substrate; $\sigma_{h}$ is the conductivity of conductive filler; $\sigma_{m}$ is the conductivity of the composite; τ is the percolation coefficient of composites.

Because the conductivity of the conductive particles is far greater than that of the rubber matrix, $\sigma_{1}=0$, To ρm=1σm, ρh=1σh, and Equation (1) can be simplified to:

(2) $$\rho_{m}=\rho_{h}{\left(\frac{1-\phi_{c}}{\phi -\phi_{c}}\right)}^{\tau }$$

Obtained by Ohm’s law, the resistance R of the conductive composite material is:

(3) $$R=\rho_{m}\kappa$$

where κ=l/S, κ is geometric coefficient of resistance; l is thickness of conductive composite, S is cross-sectional area, $\rho_{m}$ is the resistivity of conductive silicon.

When the applied stress is between 0 and 2 MPa, the change of Poisson’s ratio υ and Young’s modulus E is small. P is the pressure of the material. Conductive silicone rubber can be considered as ideal elastomer:

(4) $$\frac{1}{\kappa }\frac{\partial \partial }{\partial P}=-\frac{1+2\upsilon }{E}$$

Integrating Equation (4):

(5) $$\kappa =\kappa_{0}\exp\left(-\frac{1+2\upsilon }{E}\right)$$

where $\kappa_{0}$ is the geometric coefficient of resistance without load. The volume fraction of the conductive filler is $\phi =V_{1}/V_{m}$, where $V_{1}$ is the volume of the conductive filler, $V_{m}$ is the total volume of the silicone material, and its differential can be:

(6) $$\frac{1}{\phi }\frac{\partial \phi }{\partial P}=\frac{1}{V_{1}}\frac{V_{1}}{\partial P}-\frac{1}{V_{m}}\frac{V_{m}}{\partial P}$$

The metal filler can be seen as a rigid body, its volume change is approximately zero, that is $\frac{1}{V_{1}}\frac{V_{1}}{\partial P}=0$, then Equation (6) can be simplified to:

(7) $$\frac{1}{\phi }\frac{\partial \phi }{\partial P}=-\frac{1}{V_{m}}\frac{V_{m}}{\partial P}$$

The conductive silicone rubber is ideal elastomer $\frac{1}{V_{m}}\frac{\partial V_{m}}{\partial P}=\frac{2\upsilon -1}{E}$, substituted into Equation (7):

(8) $$\frac{1}{\phi }\frac{\partial \phi }{\partial P}=\frac{1-2\upsilon }{E}$$

Integrating gives Equation (8):

(9) $$\phi =\phi_{0}exp\left(\frac{1-2\upsilon }{E}P\right)$$

where, $\phi_{0}$ is the volume fraction of metal under no external force, so substituting Equation (9) into Equation (2):

(10) $$\rho_{m}=\rho_{h}{\left(1-\phi_{c}\right)}^{\tau }{\left[\phi_{0}exp\frac{1-2\upsilon }{E}P-\phi_{c}\right]}^{-\tau }$$

where P=F/S=σ=Eε, θ=(1−2υ)·ε, ε is the strain of conductive silicone rubber under stress. After further simplifying the formula we can get:

(11) $$\rho_{m}=\rho_{h}{\left(\frac{1-\phi_{c}}{\phi_{0}e^{\theta }-\phi_{c}}\right)}^{\tau }$$

where θ is volume strain. Equation (11) describes the relationship between the resistivity and volume strain of conductive silicone rubber.

### 2.2. Current Field Model

**Figure 2:**
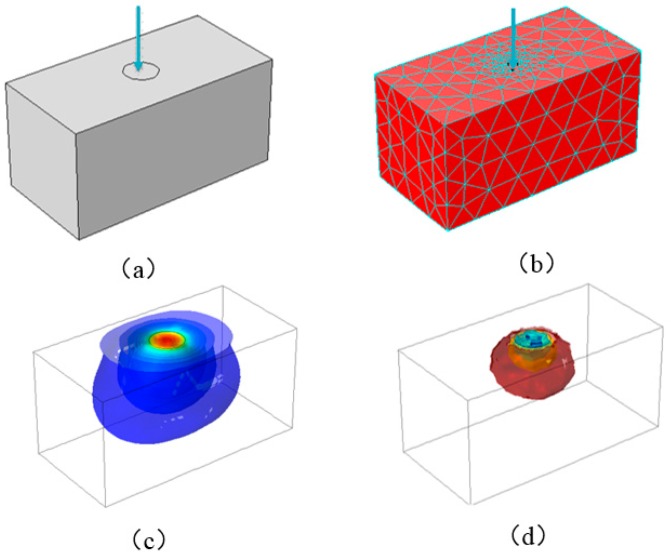
(**a**) The schematic diagram of a conductive silicone rubber under the external force; (**b**) The finite element model of conducting silicon rubber under the external force; (**c**) The three-dimensional stress isometric surface of the conductive silicon rubber under the external force. the blue area in the (**c**) represents the stress equivalent plane; (**d**) The conductivity isometric surface (The red area in the (**d**) represents equivalent surface of conductivity).

The resistivity of conductive silicone rubber is related to its volumetric strain. Suppose a piece of conductive rubber is subjected to the pressure shown in Figure 2a. Figure 2b–d are the three-dimensional stress distribution obtained by finite element analysis, It should be mentioned that the deformation of rubber usually be modeled by large deformation computational methods, such as nonlinear FEM and nonlinear discrete models [40,41]. Figure 2a is a schematic diagram of a conductive silicone rubber under the external force. Figure 2b is a finite element model of conducting silicon rubber under the external force. Figure 2c is the three-dimensional stress isometric surface of the conductive silicon rubber under the external force. Figure 2d is the conductivity isometric surface.

Therefore, if the resistivity distribution of the conductive silicone rubber can be obtained, the stress distribution can be reflected, and the direction and magnitude of the force can be analyzed. If a constant current source is applied to a variety of resistivity conductors, the voltage difference between any two points on the conductor surface can be measured. Through a series of potential differences, we should theoretically be able to reverse the distribution of internal resistivity. In order to achieve the above goal, we need to study the model of electric potential distribution in constant current excitation.

To solve the potential distribution of conductive silicon rubber under constant current excitation, which can be considered as the solution of steady field, one of the core implications of stable field is that the transmission time of current from one point to another can be ignored. Because there is no excitation source in conductive silicon rubber, the divergence of current in the solution domain is zero. Any point of the conductive silica satisfies the following formula:

(12) $$\{\begin{array}{c} \nabla \cdot \left(\sigma \cdot \nabla \phi \right)=0\\ \int_{E^{+}}\sigma \cdot \frac{\partial \phi }{\partial \vec{n}}d\vec{s}=+I\\ \int_{E^{-}}\sigma \cdot \frac{\partial \phi }{\partial \vec{n}}d\vec{s}=-I \end{array}$$

where $\vec{n}$ is normal vector on the outer boundary, E is electric-field strength, σ is electronic conductivity, ϕ is potential distribution in the field, I is excitation current.

After establishing the field model under the excitation of constant current source of conductive silica, the analytic expression of the potential distribution in the field can be obtained by a theoretical method and the analytical result of the measured voltage in the boundary state can be obtained. The derivation of this method is complex, and it is suitable for the calculation of the potential distribution of conductive silica gel with simple two-dimensional shape or a simple non-homogeneous state. However, the analytical solution is difficult to obtain for the complex non homogeneous field with irregular shape and various 3-d fields. The finite element method is a general method to solve the above problems, which called grid method and discretizes the mesh in the field and boundaries. It converts the potential function in a continuous field into a set of discrete nodal potential functions. Through using the difference principle, the difference quotient of the potential function at discrete points is used instead of the partial derivative of this point, which transforms the boundary value problem into a set of algebraic equations. In the form of matrix, the finite element equations under constant current excitation of conductive silica gel are obtained:

(13) $${\left[K\right]}_{n\times n}\times {\left[\phi \right]}_{n\times 1}={\left[P\right]}_{n\times 1}$$

where n is the number of total nodes of the field. ${\left[K\right]}_{n\times n}$ is the coefficient matrix of total electric field, which is a large, symmetric and positive definite sparse matrix. ${\left[\phi \right]}_{n\times n}$ is the voltage vector of all nodes in the field. ${\left[P\right]}_{n\times 1}$ is consisted of unit load matrix $P^{e}$ $P^{e}$ is zero for all quadrilateral elements that are not touched by all excitation sources. For the quadrilateral elements of the boundary and adjacent to the excitation element, the values of $P^{e}$ are calculated by the following formula:

(14) $$P^{e}=\int_{L}Ngdl$$

where $N=\left[N_{i},N_{j},N_{k},N_{l}\right]$, $N_{i},N_{j},N_{k},N_{l}$ are the shape functions of node i,j,k,l respectively, and the expressions are:

(15) $$\{\begin{array}{c} N_{1}=0.25\left(1-\xi \right)\left(1-\eta \right)\\ N_{2}=0.25\left(1+\xi \right)\left(1-\eta \right)\\ N_{3}=0.25\left(1+\xi \right)\left(1+\eta \right)\\ N_{4}=0.25\left(1-\xi \right)\left(1+\eta \right) \end{array}$$

### 2.3. Sensitive Field Analysis

When the conductive silicone is loaded, the conductivity in the sensitive area will change, which will cause a change in the measured voltage of the boundary. Assume that the conductivity of conductive silica gel is uniform, its conductivity is ρ. The excitation current I is applied to the *m*-th pair of electrodes, the voltage measured on the *n*th pair of the electrodes is V(m,n). When a slight change in conductivity in a certain micro domain is δρ(x,y,z), the voltage value measured on the corresponding *n*th pair of electrodes is V(m,n)+δV(m,n).

If δρ(x,y,z) is small enough, the distribution of the potential equipotential surface in the sensitive field can be considered to be unchanged, then the change of measurement voltage is proportional to the conductivity. The ratio constant $S_{m,n,x,y,z}$ is defined as the sensitivity coefficient of conductive silica gel:

(16) $$S_{m,n,x,y,z}=\frac{\delta V\left(m,n\right)}{\delta \rho \left(x,y,z\right)}$$

where m is the sequence number of the excitation electrode, n is the sequence number of the measuring electrode, and x,y,z is the coordinate value of the sensitivity coefficient formula. The calculation of sensitivity coefficient can be converted into integral formula by compensation principle of Gese-Lowitz. Then the finite element method is used to calculate the following formula:

(17) $$S_{i,j}=-\frac{1}{2V_{ej}I_{n}I_{m}}{\left[\phi_{mj}^{k}\right]}^{T}\left[Y_{j}\right]\left[\phi_{nj}^{k}\right]$$

where subscript i represents the *i*-th independent measurement. $e_{j}$ represents the *j*-th element $\phi_{m}$, $\phi_{n}$ are the nodal potential distribution in the uniform conductivity distribution field. When excited current, $I_{m}$, $I_{n}$ are applied to the *m*-th and *n*-th pair electrode, respectively. $V_{ej}$ is the volume of the *j*-th elements. $\phi_{mj}^{k}$, $\phi_{nj}^{k}$ is the potential of k nodes of the element $e_{j}$. $Y_{j}$ is the finite element coefficient matrix of element $e_{j}$.

### 2.4. Stress Field Inversion

The distribution of resistivity of conductive silica gel can be obtained by the load state of conductive silicone. If we can obtain the conductive silica gel conductivity distribution, the conductive silica loaded state can naturally be reversed. Therefore, the problem of stress field inversion is converted into solving the distribution of conductivity of conductive silica gel under the condition of the electrode potential difference of the known boundary measurement.

In the ideal noise-free conditions, the voltage of the measured electrode under the excitation of the constant current source can be expressed as a uniform operator equation. As shown in the following equation:

(18) V=F(ρ)

where V is the potential difference of the electrode pair, F is forward operator, and ρ is the conductivity distribution in the conductive silica gel. After Equation (18) is linearized, the relationship between the voltage difference ΔV and the conductivity difference Δρ can be obtained by the Taylor expansion method:

(19) $$\Delta V=\frac{\partial F}{\partial \sigma }\left(\Delta \rho \right)+O\left({\left(\Delta \rho \right)}^{2}\right)$$

If Δρ is very small, the higher order term in Equation (19) can be ignored, then Equation (19) can be reduced to:

(20) ΔV=SΔρ

where *S* is called the sensitivity matrix, assuming that the sensitive field is divided into *m* elements, a total of *n* alone measured data. After the Equation (20) is discretized and normalized, the following equation can be obtained:

(21) U=S×G

where U is the measured voltage vector after dimension normalization, S is the sensitivity matrix after n×m dimension normalization, and G is the conductivity distribution vector after m×1 dimension normalization. The problem of conductivity reconstruction is the key to the reversal of stress field of conductive silica gel. The problem can be classified mathematically in the process of reversing the problem Equation (18):

(22) $$\rho =F^{-1}\left(V\right)$$

The approximate linear solution of the inverse problem can be expressed as:

(23) $$G=S^{-1}\cdot U$$

**Figure 3:**
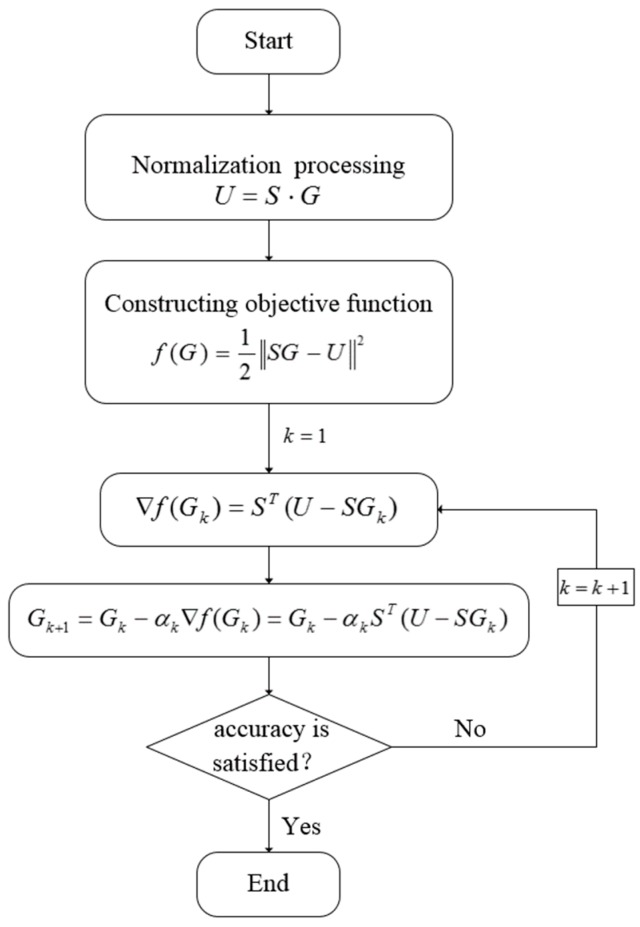
Landweber inversion algorithm flow.

According to the voltage value of the electrode pairs, the stress distribution of the conductive rubber is equivalent to the resistivity distribution of the reversal conductive rubber, which is equivalent to solving the normalized gray value (it is proportional to that conductivity distribution of the material) G. The gray value G can be obtain according to an inversion algorithm; Landweber is a common iterative algorithm with the following iterative formula:

(24) $$G_{k+1}=G_{k}+\alpha_{k}S^{T}\left(U-SG_{k}\right)$$

where k represents the number of iterations, $G_{k}$ represents the image gray value at the time of the kth iteration, $\alpha_{k}$ represents the gain factor and $\alpha_{k}=\frac{∥S^{T}Q_{k}∥{}^{2}}{∥SS^{T}Q_{k}∥{}^{2}},\;Q_{k}=U-SG_{k}$;

The Landweber inversion algorithm is shown in Figure 3:

(1) The sensitive field is divided into a plurality of elements by finite element subdivision, and the sensitive field is normalized;
(2) Construct precision objective function. Image reconstruction according to the voltage value of the measurement electrode is equivalent to solving the normalized gray value G. This problem can be evolved into an optimization problem, assuming that any given normalized gray value G, according to U=S×G, a theoretical calculated voltage value, then solve the gray value can be attributed to the optimization problem of the objective function;
(3) Set the iteration coefficient k; Update and calculating $G_{k}$ accord to an iteration formula;
(4) Judge whether f(G) reach a specified precision threshold value or not, and if so, taking $G_{k}$ as a final gray value; If the specified precision threshold is not reached, $G_{k+1}=G_{k}$ until the precision requirements are met.

According to the relationship between conductivity and volume strain, the distribution of volume strain can be obtained. And then the stress distribution can be obtained.

**Figure 4:**
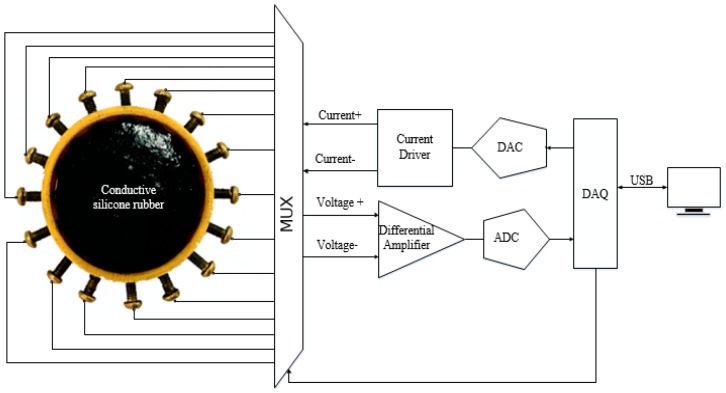
Schematic diagram of conductivity distribution measurement device.

The measuring device of internal stress distribution of conductive silica gel is shown in Figure 4. A series of conductive electrodes are arranged on the side of conductive silica gel. The conductive electrode is connected with a conductive adhesive for conductive rubber.

The conductive electrode is connected with a multiplex switch circuit which is controlled by a computer. A constant current source is injected into the conductive rubber through the electrode by a preset excitation sequence. The other electrode acts as the voltage collector to collect the voltage value of the adjacent electrodes. The voltage signal is input to the data acquisition card through the differential amplifier circuit, which will store the collected voltage signals to the computer according to certain rules .The rule refers to the order selection rules of constant current source excitation and measurement of the electrode affixed with conductive silica gel. Taking the circular silica disk in the illustration as an example, the sequence of excitation and measurement is: first, No.1 and No.2 electrodes on constant current *I*, measuring voltage of 2–3, 3–4, …, 15–16 on measurement circuit. The second, No.2 and No.3 electrodes connect constant current source *I* and measurement circuit to measure the voltage of 3–4, 4–5, …, 1–2, respectively.

By analogy, the excitation and measurement of all electrodes are completed. According to the measured voltage value, the gray distribution of representative resistivity can be obtained, and the stress field distribution can be obtained. The device can be used as a sensing device for plane stress, such as sensing the stress distribution of human body pressure [42].

## 3. Results and Discussion

### 3.1. The Pressure Distribution in the Conductive Silicone Pad

**Figure 5:**
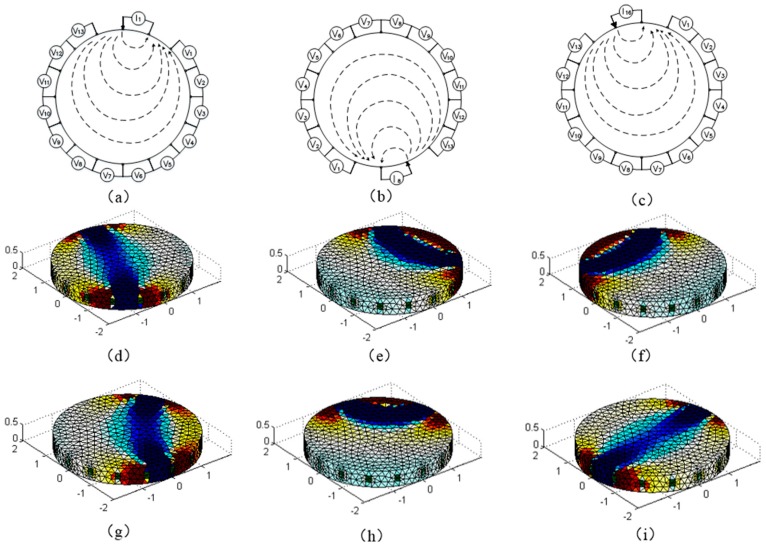
(**a**) The way of measuring the voltage when the 1,2 electrode are electrified; (**b**) The way of measuring the voltage when the 8,9 electrode are electrified; (**c**) The way of measuring the voltage when the 16,1 electrode are electrified; (**d**–**i**) The result of the sensitive distribution at 8,16,24,32,40,48 measurements (The blue area in (**d**–**i**) represents the sensitive area of the disc conductive silicone rubber).

**Figure A1:**
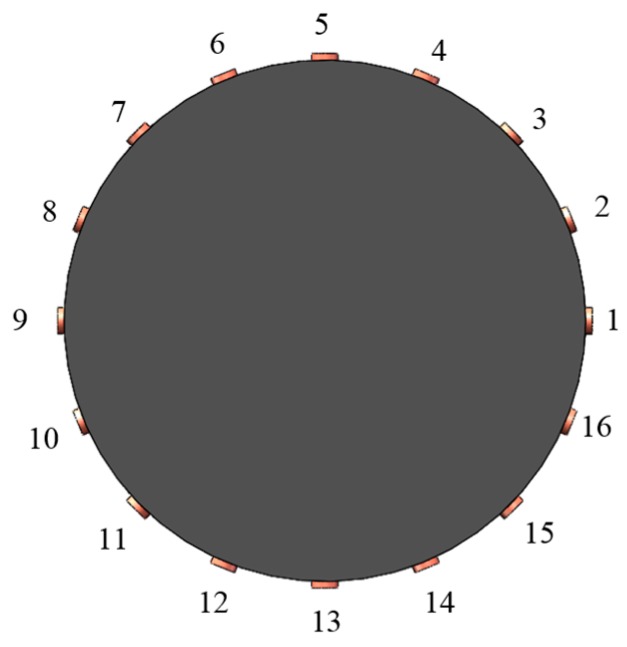
The arrangement of the single-row electrodes uniformly arranged counterclockwise along the circumferential direction of the conductive silicone rubber.

The characteristics of changes of conductivity of the conductive silicone film can be used to detect the force distribution of the surface, and can be used in the field of electronic skin sensing and other fields [43]. The traditional method is to make the conductive silicone into the form of the array, which makes the circuit design complex, the cost high and its resolution be related to the number of sensors in the array. Based on the current field characteristics of the conductive silicone film under the action of the constant current source, we can invert the conductivity distribution of the conductive silicone film through a few electrodes, and then reconstruct the pressure distribution state of its surface. Figure 5 is a voltage measurement method and a sensitive field distribution of a disk-shaped conductive silicone rubber. The thickness of the conductive silica is 5 mm, and the radius is 20 mm. Figure 5a–c show the schematic of the adjacent method injecting electrical current and measuring voltage potentials via multiplexing. The 16 electrodes were evenly arranged on the outer periphery of the circular silicone pad, and two of the electrodes were used as excitation electrodes for the constant current source, and the remaining electrodes were used as the measuring electrodes. The electrode arrangement of the disc-shaped conductive silicone rubber is shown in Figure A1 in Appendix A. The excitation electrode and the measuring electrode are rotated according to the rules of the adjacent excitation. Taking 16 electrodes as an example, 208 sets of measurement voltage data (16 × (16 − 3) = 208) can be generated by alternating the measurement and excitation electrodes. The sequence of rotation of the excitation and measurement electrodes is shown in Appendix B. The distribution of the conductivity of the conductive silica gel can be obtained by solving the Equation (23) by means of iterative optimization. The key step in solving Equation (23) is to obtain the distribution of the sensitivity coefficient of the conductive silica gel in different excitation modes. Figure 5d–i are the results of the sensitivity distribution of different incentive models.

**Figure 6:**
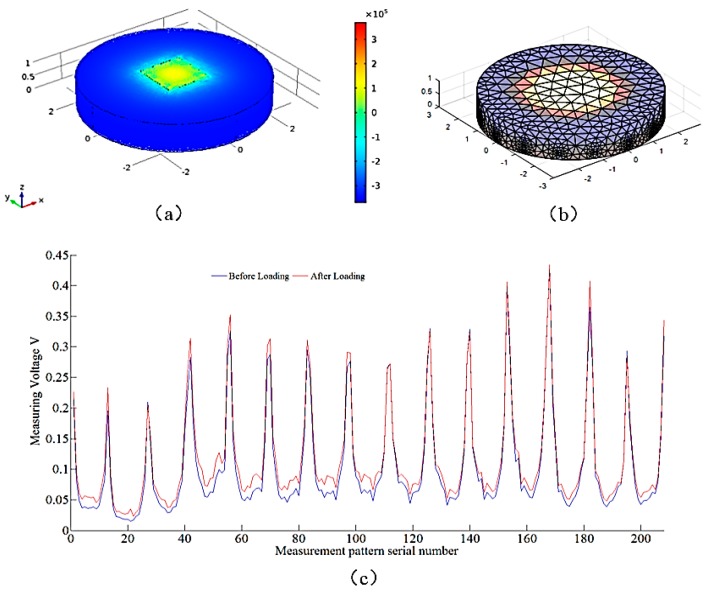
(**a**) is the results of stress distribution of finite element analysis of conductive silica disks; (**b**) is the results of resistivity inversion imaging of conductive silica pad; (**c**) is the voltage of the measuring electrode pair in the adjacent excitation mode of the conductive silica gel. The *x* axis is the serial number of the measurement, and the sequence number is related to the excitation mode. The *y*-axis is the voltage of the measured electrode. Its unit is volts.

Loading the force of 20 N on the conductive silicone pad, the loading surface is 10 mm × 10 mm square. Figure 6a is the results of stress distribution of conductive silica pad by using finite element analysis. Figure 6b is the result of the conductivity of the loaded conductive silica, which is based on the shape of the conductive silica and the voltage data of the measuring electrode. It can be seen that the distribution of resistivity can reflect the distribution of pressure. This means that a small amount of electrodes can be used to deduce the pressure distribution of the conductive silica surface. Figure 6c is a voltage value of an electrically conductive silicone disc measured at a load of not loaded and loaded with 20 N.

The *x* axis is the serial number of the measurement, and the sequence number is related to the excitation mode. The *y*-axis is the voltage of the measured electrode, its unit is volts. This suggests that the results of this study can be applied to the field of measurement of the distribution of forces such as electronic skin to reduce the cost of conventional dot-matrix force sensitive sensors.

### 3.2. Inversion Results of Three Dimensional Stress Field

**Figure 7:**
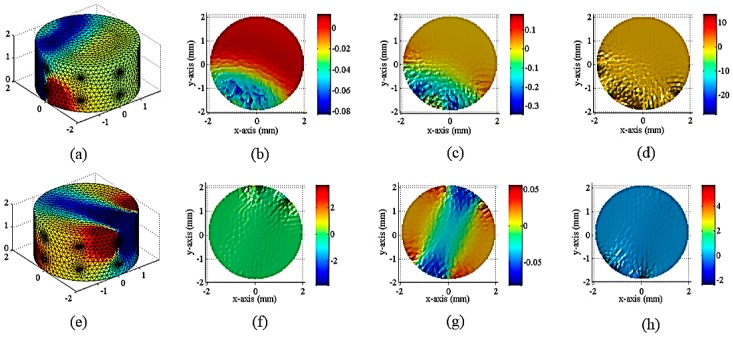
(**a**) The result of the sensitivity distribution of the cylindrical conductive silicone rubber at the 5th measurement; (**b**–**d**) The sensitivity profile at 0.5, 1.0 and 1.5 cm from the bottom surface of the cylindrical conductive silicone rubber at the 5th measurement respectively; (**e**) The result of the sensitivity distribution of the cylindrical conductive silicone rubber at the100th measurement; (**f**–**h**) The sensitivity profile at 0.5, 1.0 and 1.5 cm from the bottom surface of the cylindrical conductive silicone rubber at the 100th measurement, respectively.

**Figure 8:**
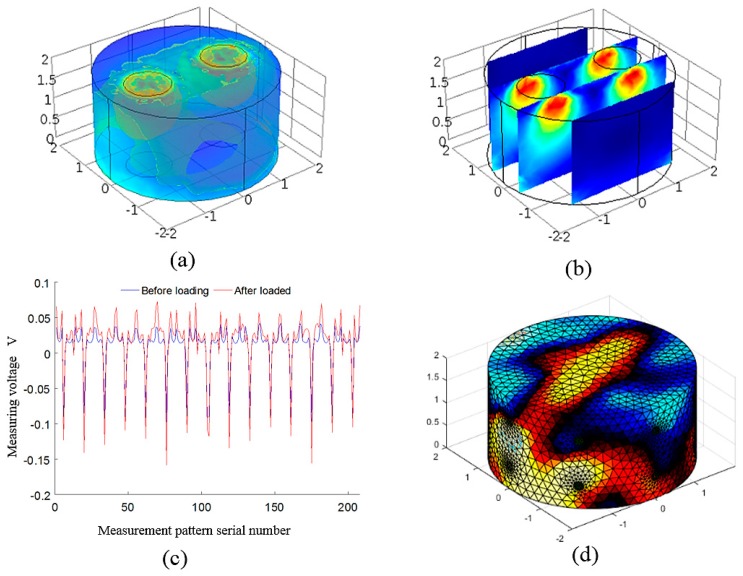
(**a**) The isosurface of stress three-dimensional distribution of the cylindrical conductive silica obtained by finite element analysis under the action of a 20 N force. Different colors in the (**a**) represent different equivalent surfaces; (**b**) the sectional view of the stress distribution in the vertical direction (The red area in the (**b**) represent a region of high stress in a cylindrical conducting silicone rubber); (**c**) The voltage value of the measuring electrode under load 20 N force and no load force. The *x* axis represents the measured sequence number and the *y* axis represents the voltage value of the measuring electrode, and its unit is volts. The red line represents the measured voltage value after loading, and the blue line represents the measured voltage without load; (**d**) The result of the three-dimensional distribution inversion of the conductivity under a load of 20 N in accordance with the measured voltage (The red area at the top in the (**d**) represents a region of high stress in a cylindrical conducting silicone rubber).

**Figure 9:**
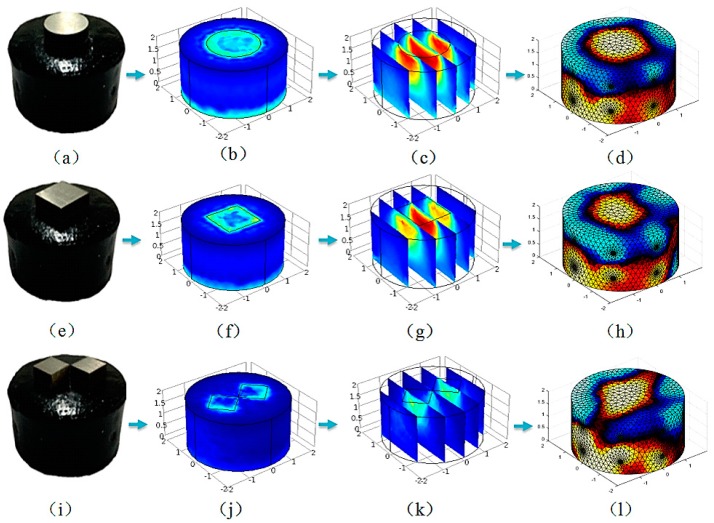
(**a**) A loading example placing a loaded head with a circular radius of 10 mm on the conductive silicone upper surface; (**b**) The result of finite element analysis of a cylindrical conductive silicone rubber under stress in a circular region; (**c**) The sectional view of the stress distribution in the vertical direction (The red region in the (**c**) represents the region with the greatest stress); (**d**) The result of the inversion of the conductivity three-dimensional distribution under the circular region load (The red area at the top in the (**d**) represents a region of high stress in a cylindrical conducting silicone rubber); (**e**) The loading example placing a loaded head with a square side length of 15 mm on the conductive silicone upper surface; (**f**) The result of finite element analysis of a cylindrical conductive silicone rubber under stress in a square region; (**g**) The sectional view of the stress distribution in the vertical direction (The red region in the (**g**) represents the region with the greatest stress); (**h**) The result of the inversion of the conductivity three-dimensional distribution under the square region load (The red area at the top in the (**h**) represents a region of high stress in a cylindrical conducting silicone rubber); (**i**) A loading example placing a loaded head with two squares of 8 mm × 8 mm on the conductive silicone upper surface; (**j**) The result of finite element analysis of a cylindrical conductive silicone rubber under stress in two square region; (**k**) A sectional view of the stress distribution in the vertical direction (The yellow region in the (**k**) represents the region with the greatest stress); (**l**) The result of the inversion of the conductivity three-dimensional distribution under two square region load (The red area at the top in the (**h**) represents a region of high stress in a cylindrical conducting silicone rubber).

The perception ability of the stress distribution was measured by using a pressure test with a cylindrical three-dimensional piece of conductive silica (radius 20 mm, height 20 mm). Figure 7 is a sensitivity profile of a cylindrical conductive silicone rubber, and Figure 8 and Figure 9 show the finite element simulation and the three-dimensional distribution of electrical conductivity of cylindrical conductive silica under different load conditions. Compared with the stress distribution obtained by finite element analysis, its potential application value is proved. The excitation and measurement electrodes are arranged on the sides of the cylinder and in two layers. Each layer is evenly arranged with eight electrodes that are evenly arranged in the circumferential direction (Figure 7). The excitation electrode and the measuring electrode are rotated according to the rules of the adjacent excitation. With 16 electrodes as an example, 208 sets of voltage data (16 × (16 − 3) = 208) can be measured by electrode rotation. The specific order of measurement is shown in the (Appendix C). Figure 7 is the result of sensitivity distribution of different incentive models. The sensitivity coefficient is related to the excitation mode of the electrode. Figure 7a is a sensitivity distribution of a cylindrical conductive silicone rubber at the time of the fifth measurement. Figure 7b–d show the sensitivity distribution of the cylindrical conductive silicone rubber at height 0.5, 1.0, and 1.5 cm from the bottom surface of the cylindrical conductive silicone rubber. Figure 7e–h are a sensitivity distribution of a cylindrical conductive silicone rubber at the time of the 100th measurement. When the sensitivity matrix in each excitation mode is calculated by the finite element method, the distribution of the conductivity of the conductive silica can be reversed according to the measured voltage value of the measuring electrode.

Figure 8 shows the results of the first loading experiment of the cylindrical conductive silica. The experiment was carried out by placing two convex heads with a diameter of 8 mm on the upper end of the cylindrical conductive silicon, and compared the three-dimensional distribution of conductivity generated by the inversion and the stress generated by the finite element. Figure 8a shows the three-dimensional stress distribution of the cylindrical conductive silica obtained by finite element analysis under the action of a 20 N force. Figure 8b shows the slice of the internal stress distribution in the *z*-axis direction. Figure 8c is the voltage value of the measuring electrode under load 20 N force and no load force. The *x* axis represents the measured sequence number, and the *y* axis represents the voltage value of the measuring electrode, and its units are volts. The red line represents the measured voltage value after loading, and the blue line represents the measured voltage without load. Figure 8d is the result of the three-dimensional distribution inversion of the conductivity under a load of 20 N in accordance with the measured voltage.

Figure 9a shows a loading example that placing a loaded head with a circular radius of 10 mm on the conductive silicone upper surface. Figure 9e is the loaded head of a square with a size 15 mm × 15 mm, and Figure 9i is the loaded head with two squares of 8 mm × 8 mm, on which the loading pressure is 20 N. The child of each of Figure 9 are respectively from left to right: the sample of conducting silica, the shape of the loaded head, the 3D stress distribution of the conductive silica obtained by finite element analysis, Z-direction slice display of three-dimensional stress distribution, and the inversion results of 3D conductivity distribution of conductive silica.

### 3.3. Demonstration of Force Direction Detection Device

**Figure 10:**
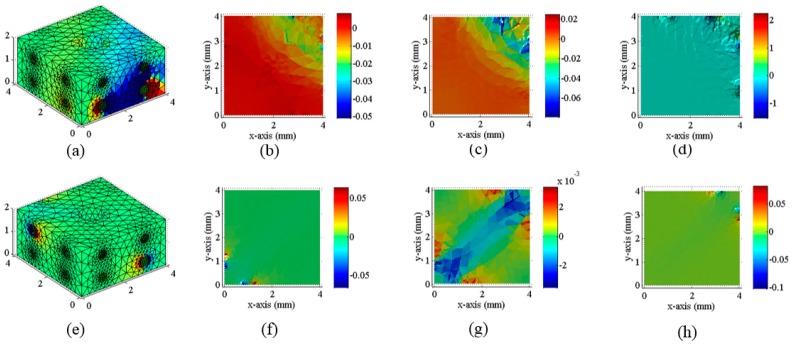
(**a**) The result of the sensitivity distribution of the rectangular conductive silicone rubber with a hemispherical hole at the top at the 5th measurement; (**b**–**d**) The sensitivity profile at 0.5, 1.0 and 1.5 cm from the bottom surface of the rectangular conductive silicone rubber at the 5th measurement, respectively; (**e**) The result of the sensitivity distribution of the rectangular conductive silicone rubber with a hemispherical hole at the top at the 100th measurement; (**f**–**h**) The sensitivity profile at 0.5, 1.0 and 1.5 cm from the bottom surface of the rectangular conductive silicone rubber at the 100th measurement, respectively.

The proposed sensor has a great potential to be used as a force directional receptor of the joint of the machine because it can detect the stress distribution of different direction forces. The prototype of the sensor is made as follows: a cuboid with 40 mm × 40 mm × 20 mm is made with electrically conductive silicone. The center of the upper surface has a hemispherical concave hole, which can be seen as a spherical joint. Through the joint hinged bar, a force of 20 N is applied in the axial direction of the pole. The measuring electrode and the driving electrode shall be arranged in two layers with each side arranged in two layers, with a total of 16 electrodes arranged on each side. A total of 208 voltage measurement data can be obtained. Figure 10 shows the results of the sensitivity distribution of different incentive models. Figure 10a is a sensitivity distribution of a rectangular conductive silicone rubber at the time of the fifth measurement. Figure 10b–d show the sensitivity distribution of the rectangular conductive silicone rubber at heights of 0.5, 1.0 and 1.5 cm from the bottom of the rectangular conductive silicone rubber. Figure 10e–h show the sensitivity distribution of a rectangular conductive silicone rubber at the time of the 100th measurement.

**Figure 11:**
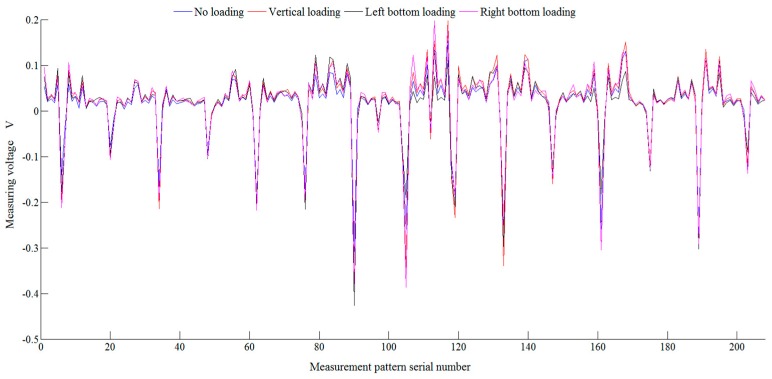
Measured voltage values of rectangular conductive silicone rubber under different direction forces. The *x* axis represents the measured sequence number, and the *y* axis represents the voltage value of the measuring electrode, in volts. The blue line represents the measured voltage with no load. The black line represents the measured voltage when the angle between the load direction and the horizontal direction is 30 degrees; the red line represents the measured voltage when the angle is 90 degrees; the purple line represents the measured voltage when the angle between the load direction and the horizontal direction is 120 degrees.

Figure 11 is a measurement voltage value of a rectangular conductive silicone rubber under the action of a force of 20 N in different directions. The *x* axis represents the measured sequence number, and the *y* axis represents the voltage value of the measuring electrode, in volts. The blue line represents the measured voltage under no load. The black line represents the measured voltage when the angle between the load direction and the horizontal direction is 30 degrees. The red line represents the measured voltage when that is 90 degrees. The purple line represents the measured voltage when the angle between the load direction and the horizontal direction is 120 degrees.

**Figure 12:**
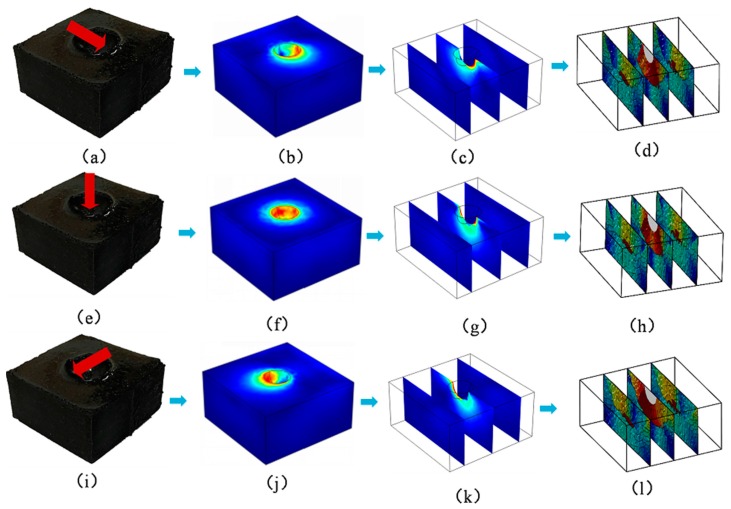
The results of finite element analysis under various angles of hinged rods, the slices of stress, and the inversion results of resistivity distribution, respectively (The red region in the (**b**–**d**), (**f**–**h**), (**j**–**k**) indicate the region with greater stress on the conductive silicone rubber); (**a**) The loading example of the rectangular conductive silicone rubber with a hemispherical hole at the top under the stress tilted to the right; (**b**) The finite element analysis result of a rectangular conductive silicone rubber under the stress tilted to the right; (**c**) The sectional view of the stress distribution; (**d**) The sectional view of the three-dimensional distribution inversion of the electrical conductivity under the stress tilted to the right; (**e**) The loading example of the rectangular conductive silicone rubber with a hemispherical hole at the top under vertical stress; (**f**) The result of finite element analysis of a rectangular conductive silicone rubber under the vertical stress; (**g**) The sectional view of the stress distribution; (**h**) The sectional view of the three-dimensional distribution inversion of the electrical conductivity under the vertical stress; (**i**) A loading example of the rectangular conductive silicone rubber with a hemispherical hole at the top under the stress tilted to the left; (**j**) The finite element analysis result of a rectangular conductive silicone rubber under the stress tilted to the left; (**k**) The sectional view of the stress distribution; (**l**) The sectional view of the inversion of the conductivity three-dimensional distribution under the stress tilted to the left.

**Figure 13:**
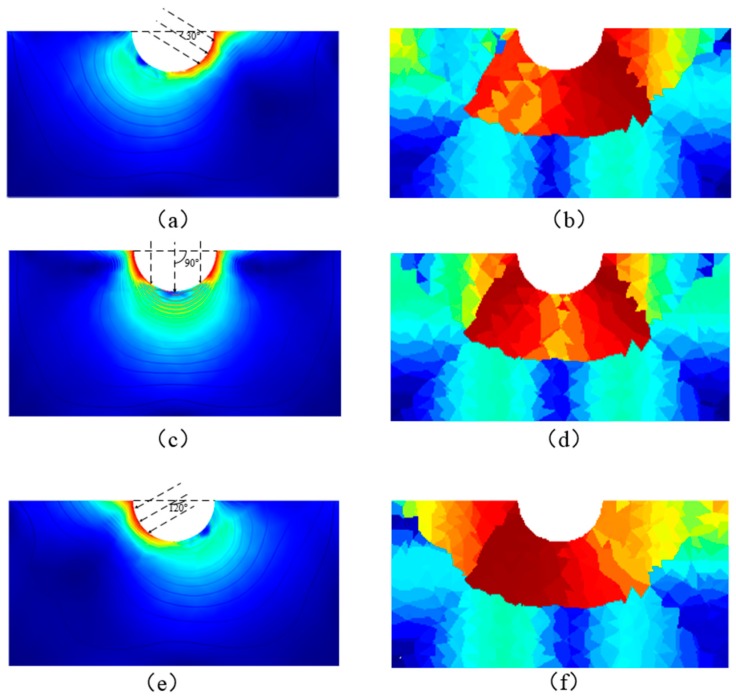
The result of the finite element analysis of the middle section and the inversion result of conductivity distribution when the rectangular conductive silicone rubber with hemispherical grooves on the top is cut along the vertical direction; (**a**) A finite-element analysis of the middle section of a rectangular conducting silicon rubber with a hemispherical groove at the top, when the hemispherical concave surface is loaded along the direction of 30 degrees; (**b**) A slice of the inversion result of the conductivity distribution in the middle section of a rectangular conductive silicone rubber; (**c**) A finite-element analysis of the middle section of a rectangular conducting silicon rubber with a hemispherical groove at the top, when the hemispherical concave surface is loaded along the direction of 90 degrees.; (**d**) A slice of the inversion result of the conductivity distribution in the middle section of a rectangular conductive silicone rubber; (**e**) A finite-element analysis of the middle section of a rectangular conducting silicon rubber with a hemispherical groove at the top, when the hemispherical concave surface is loaded along the direction of 90 degrees; (**f**) A slice of the inversion result of the conductivity distribution in the middle section of a rectangular conductive silicone rubber. (The red region in the (**a**–**f**) indicate the region with greater stress on the conductive silicone rubber)

Figure 12 and Figure 13 are the three-dimensional stress distribution obtained by finite element analysis, the slice of stress, and the inversion results of conductivity at different force angles, respectively.

## 4. Materials and Methods

### 4.1. Preparation of Conductive Silicon Rubber

**Figure 14:**
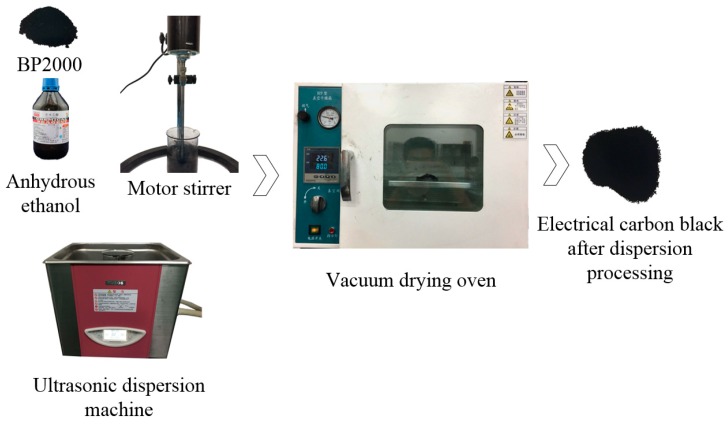
The dispersion process of conductive carbon black. The conductive carbon black is dispersed, stirred and dried to become uniformly distributed and non-agglomerated carbon black.

We use conductive silicone rubber as the pressure-sensitive sensing material, which be made by mixing conductive carbon black (BP2000, CABOT, Boston, MA, USA) and silicone rubber. The preparation process is as follows:

Step 1: The conductive carbon black (BP2000) was dispersed: The conductive carbon black was mixed with anhydrous ethanol and stirred for 10 min by electric mixer. It was dispersed for 30 min by ultrasonic dispersion machine, and dried for 3 h in vacuum drying oven (60 °C, 0.08 MPa), Finally, we obtain a dispersed uniformly conductive carbon black. Figure 14 is the dispersion process of conductive carbon black.

Step 2: Petroleum ether (The quantity is 10 times that of the conductive carbon black) is mixed with Si-69 coupling agent (the mass is 5% of the silicone rubber). The conductive carbon black with 10% mass fraction was added, stirred for 10 min by electric mixer, and dispersed for 30 min by ultrasonic dispersion machine.

Step 3: The 107 silicone rubber was added to the mixture, and stirred for 5 min by electric mixer, and dispersed for 30 min by ultrasonic cleaning machine to distribute the conductive carbon black evenly in the silicone rubber. In order to accelerate the reaction, tetraethyl orthosilicate (7% mass fraction) and dibutyltin dilaurate (5% mass fraction) were added.

**Figure 15:**
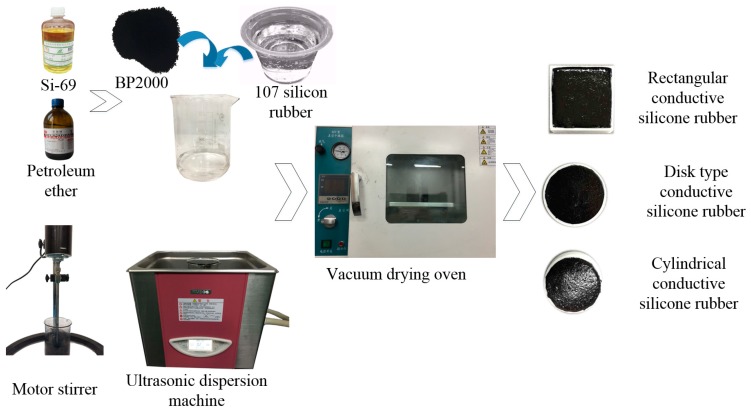
The preparation details of the conductive silicone rubber. The dispersed conductive carbon black is mixed with the silicone rubber. The conductive silicone rubber is vulcanized in a vacuum environment.

Step 4: Put the mixture into a vacuum drying oven and vacuum for 5 min at room temperature. The resulting viscous mixture is poured into a different mold to allow it to be completely cured for 24 h and made into circular, cylinder, rectangular conductive silicone rubber. Figure 15 shows the preparation details of the conductive silicone rubber.

### 4.2. Measuring Device

In order to evaluate the effectiveness and accuracy of the proposed inversion method for the internal stress distribution of conductive silica gel, it needs to be tested by experiments. So far, however, no method has been found to measure the stress distribution of non-transparent non-metallic materials. Magnetic measurements are often used to measure the residual stress of metal. The principle of magnetic measurement is to determine the magnitude and direction of residual stress by measuring the change of permeability of ferromagnetic materials under the action of internal stress.

The material of force-sensitive sensing material used in this paper is conductive silica gel. It is not a ferromagnetic material, so it cannot be used to measure the three-dimensional stress distribution inside the material by magnetic measurements. In order to evaluate the correctness of the method described in this paper, the stress distribution of conductive silica gel can only be obtained by finite element analysis. The key of finite element analysis is to obtain the mechanical constitutive parameters of conductive silica gel, such as elastic modulus, young’s modulus, compression modulus, shear modulus and so on. Then we can get the three-dimensional stress distribution of conductive silica gel by finite element analysis.

#### 4.2.1. Measurement of Physical Parameters 

**Figure 16:**
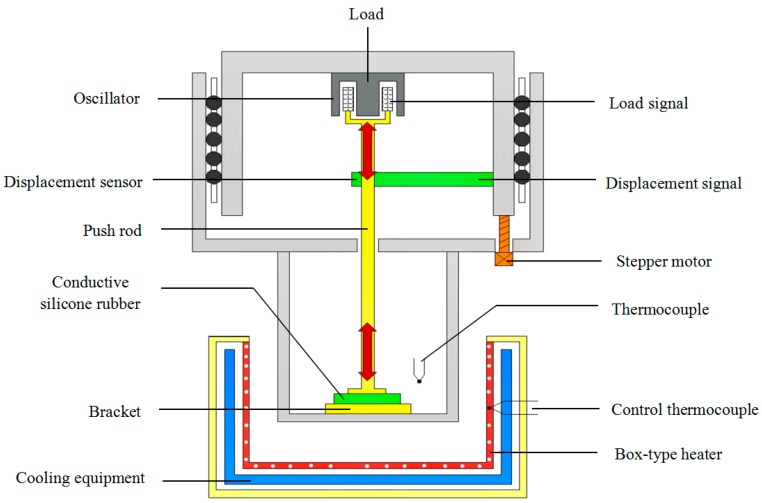
The measurement principle of DMA. The mechanical properties of the material were obtained by measuring the strain of the material under different pressures.

**Figure 17:**
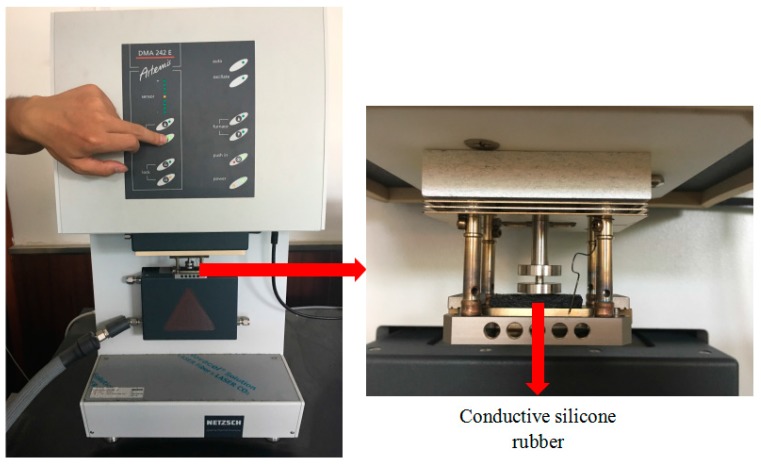
The real live-action of the conductive silica gel mechanics test.

In order to observe and record the mechanical parameters of conductive silica gel, the conductive silicone rubber was cut into the sample of 30×10×5 mm^3^ specification. And the mechanical parameters of conductive silicone rubber were determined in the dynamic thermal analyzer (DMA 242, NETZSCH, Bavaria, Germany), which is a periodic oscillation force applied to the conductive silica gel sample under certain temperature control program. The corresponding deformation amplitude and hysteresis of conductive silica gel are measured to calculate and obtain the relevant characteristic parameters such as elastic modulus, loss factor and so on. Figure 16 and Figure 17 are respectively the measurement principle of DMA and a real live-action of the conductive silica gel mechanics test.

#### 4.2.2. Voltage Measurement between Conductive Silica Electrodes 

**Figure 18:**
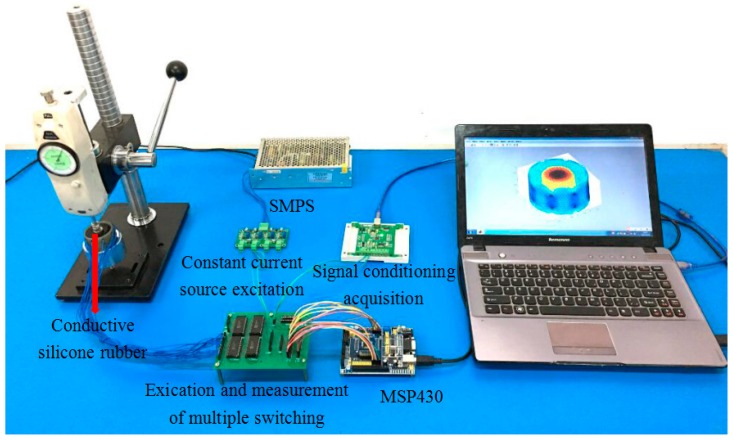
The voltage measuring device for conductive silicone rubber.

Sixteen electrodes that form a copper bolt with a diameter of 3 mm were evenly arranged around the conductive silicone rubber. In order to reduce the contact resistance at the measurement, we embed the electrodes inside the conductive rubber. The measurement of the voltage between the electrodes is as follows: the multi-channel selection module for excitation and measurement is connected to the conductive silicone rubber electrode. First, the microcontroller sends the control signal to the incentive multiple selection module. According to the preset excitation and measurement mode, the microcontroller unit takes turns to apply the excitation current of 8 mA to a pair of electrodes, and the sensitive field is established within the conductive silicone rubber. Then the microcontroller unit sends out the signal to the signal acquisition module. According to the given method, the voltage between the electrodes is measured, and the sampling frequency is 1 kHz. Figure 18 is an electrode voltage measurement device around the conductive silicone rubber.

## 5. Conclusions

In this paper, a three-dimensional visualization method of silica gel stress field based on three-dimensional resistance tomography is proposed by using the piezoresistive effect of silica gel with conductive particles. The benefits of this approach are that the three-dimensional visualization of the internal stress of the silica can be carried out by the limited electrode on the surface of the silica gel, and the stress evolution of the conductive silica can be observed dynamically, which overcomes the shortcomings of the traditional dot matrix silica gel stress sensing device that cannot obtain the three-dimensional distribution of the stress in silica gel. Critical components of this work are the fabrication of conductive silicone composites with induced strain and a 3D resistance tomography inversion algorithm based on constant current excitation. The 3D stress distributions are computed from the voltage potentials measured at the electrodes on the boundary of the conductive silicone. This approach could be used to realize multi-directional strain distribution sensors with large coverage area and three dimensional contoured shapes. A remaining technical challenge is the enhancement of the 3D stress distribution of conductive silicone and calculation accuracy in the 3D resistance tomography inversion algorithm. The experimental results show that the proposed method can reflect the three-dimensional distribution of stress in conductive silica gel. This also implies that the method has a strong potential in the application of flexible three-dimensional force sensing.

Compared with the traditional mechanical tactile sensor, the sensor studied in this paper has the following advantages: (1) compared with the existing force sensor, the new three-dimensional stress sensor proposed in this paper has the characteristics of needing less measuring electrodes, simple structure and so on; (2) the sensor can be made into any desired shape according to the application situation, and the application scene of the sensor is thus widened; (3) the three-dimensional stress field sensing method based on electrical resistance tomography can invert the stress distribution of piezoresistive sensitive materials, and has higher time resolution and dynamic measurement of stress distribution state although the spatial resolution is low; (4) it not only can obtain that stress distribution on the surface of the sensing material, but also can obtain the stress distribution state inside the sensing material. The technique can be applied to real-time stress detection of seal rings to avoid tragedies similar to the one caused by failure of the seal of the Challenger Space Shuttle. In addition it can also be used for the perception of intelligent robots’ flexible joint three dimensional force.

## Figures and Tables

**Figure A2:**
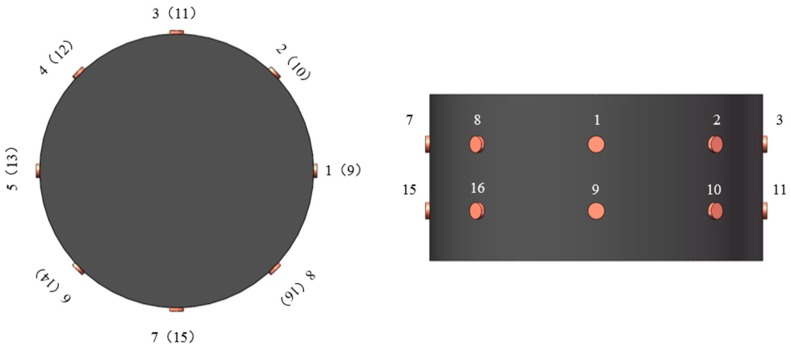
The double-layer electrode arrangement. Two layers of electrodes, and 8 electrodes in per layer and 16 electrodes in total, are uniformly arranged counterclockwise along the circumferential direction of the side surface of the conductive silicone rubber.

## Appendix A

The Appendix B and Appendix C describe the excitation and measurement scheme of single-row and double-row electrodes, respectively. The electrode arrangement is as shown in Figure A1 and Figure A2, respectively.

## Appendix B

Single layer electrode, 16 electrodes are evenly arranged counterclockwise along the side of the conductive silicone rubber. An excitation current is added to the adjacent electrodes. Voltage measurement is carried out on the adjacent electrodes, 1,2; 2,3, ⋯, electrodes are sequentially electrified, and voltages of other adjacent electrodes are measured. Each time the voltage is measured, it starts at electrodes 1 and 2, ends at electrodes 16 and 1, and the voltage on the electrode to which the excitation current is added is not measured. Specific measurement methods are shown in Appendix B.

**Table A1:** Single layer electrode measurement mode.

**Measured Sequence Number**	**Excitation Electrode Pair**	**Measuring Electrode Pair**
1	1,2	3,4
2	1,2	4,5
⋯	⋯	⋯
5	1,2	7,8
⋯	⋯	⋯
13	1,2	15,16
14	2,3	4,5
⋯	⋯	⋯
25	2,3	15,16
26	2,3	16,1
27	3,4	1,2
28	3,4	5,6
⋯	⋯	⋯
39	3,4	16,1
40	4,5	1,2
41	4,5	2,3
42	4,5	6,7
⋯	⋯	⋯
52	4,5	16,1
53	5,6	1,2
54	5,6	2,3
55	5,6	3,4
56	5,6	7,8
⋯	⋯	⋯
65	5,6	16,1
66	6,7	1,2
⋯	⋯	⋯
69	6,7	4,5
70	6,7	8,9
⋯	⋯	⋯
78	6,7	16,1
79	7,8	1,2
⋯	⋯	⋯
83	7,8	5,6
84	7,8	9,10
⋯	⋯	⋯
91	7,8	16,1
92	8,9	1,2
⋯	⋯	⋯
97	8,9	6,7
98	8,9	10,11
⋯	⋯	⋯
100	8,9	12,13
⋯	⋯	⋯
104	8,9	16,1
105	9,10	1,2
⋯	⋯	⋯
111	9,10	7,8
112	9,10	11,12
⋯	⋯	⋯
117	9,10	16,1
118	10,11	1,2
⋯	⋯	⋯
125	10,11	8,9
126	10,11	12,13
⋯	⋯	⋯
130	10,11	16,1
131	11,12	1,2
⋯	⋯	⋯
139	11,12	9,10
140	11,12	13,14
⋯	⋯	⋯
143	11,12	16,1
144	12,13	1,2
⋯	⋯	⋯
153	12,13	10,11
154	12,13	14,15
⋯	⋯	⋯
156	12,13	16,1
157	13,14	1,2
⋯	⋯	⋯
167	13,14	11,12
168	13,14	15,16
169	13,14	16,1
170	14,15	1,2
⋯	⋯	⋯
181	14,15	12,13
182	14,15	16,1
183	15,16	1,2
⋯	⋯	⋯
195	15,16	13,14
196	16,1	2,3
⋯	⋯	⋯
208	16,1	14,15

## Appendix C

Double layers electrode: Two layers of electrodes are evenly arranged along the circumferential direction in the side of the conductive silicone rubber, eight of each layer, and 16 electrodes in all. Adding excitation current to adjacent electrodes, and measuring voltages of other adjacent electrodes. Two layers of electrodes are respectively measured. First, add the exciting current to the 1,2 electrode and measure the voltage on the 3,4; 4,5⋯⋯7,8; 9,10⋯⋯16,9 electrode; Electrode 8,9 does not add excitation current nor measure its voltage. Each voltage measurement starts at electrode 1,2, ends at electrode 16,9; and does not measure the voltage on the added current electrode. Specific measurement methods are shown in Appendix C.

**Table A2:** Double layers electrode measurement mode.

**Measured Sequence Number**	**Excitation Electrode Pair**	**Measuring Electrode Pair**
1	1,2	3,4
2	1,2	4,5
⋯	⋯	⋯
5	1,2	7,8
6	1,2	9,10
⋯	⋯	⋯
12	1,2	15,16
13	1,2	16,9
14	2,3	4,5
⋯	⋯	⋯
17	2,3	7,8
18	2,3	8,1
19	2,3	9,10
⋯	⋯	⋯
26	2,3	16,9
27	3,4	1,2
28	3,4	5,6
⋯	⋯	⋯
30	3,4	7,8
31	3,4	8,1
32	3,4	9,10
⋯	⋯	⋯
39	3,4	16,9
40	4,5	1,2
41	4,5	2,3
42	4,5	6,7
43	4,5	7,8
44	4,5	8,1
45	4,5	9,10
⋯	⋯	⋯
52	4,5	16,9
53	5,6	1,2
⋯	⋯	⋯
55	5,6	3,4
56	5,6	7,8
⋯	⋯	⋯
65	5,6	16,9
66	6,7	1,2
⋯	⋯	⋯
69	6,7	4,5
70	6,7	8,1
71	6,7	9,10
⋯	⋯	⋯
78	6,7	16,9
79	7,8	1,2
⋯	⋯	⋯
83	7,8	5,6
84	7,8	9,10
⋯	⋯	⋯
91	7,8	16,9
92	8,1	2,3
⋯	⋯	⋯
96	8,1	6,7
97	8,1	9,10
⋯	⋯	⋯
100	8,1	12,13
⋯	⋯	⋯
104	8,1	16,9
105	9,10	1,2
	⋯	⋯
112	9,10	8,1
113	9,10	11,12
⋯	⋯	⋯
117	9,10	15,16
118	10,11	1,2
⋯	⋯	⋯
125	10,11	8,1
126	10,11	12,13
⋯	⋯	⋯
130	10,11	16,9
131	11,12	1,2
⋯	⋯	⋯
139	11,12	9,10
140	11,12	13,14
⋯	⋯	⋯
143	11,12	16,9
144	12,13	1,2
⋯	⋯	⋯
153	12,13	10,11
154	12,13	14,15
⋯	⋯	⋯
156	12,13	16,9
157	13,14	1,2
⋯	⋯	⋯
167	13,14	11,12
168	13,14	15,16
169	13,14	16,9
170	14,15	1,2
⋯	⋯	⋯
181	14,15	13,14
182	14,15	16,9
183	15,16	1,2
⋯	⋯	⋯
195	15,16	14,15
196	16,9	1,2
⋯	⋯	⋯
203	16,9	8,1
204	16,9	10,11
⋯	⋯	⋯
208	16,9	14,15
